# Oral Delivery of a GI-Stable Apigenin–Cyclodextrin Complex via Pectin-Coated Nanoliposomes In Situ Gel: A DoE-Optimized Targeted Colon Cancer Therapy by Modulating Gut Drug Sensitivity

**DOI:** 10.3390/gels11110873

**Published:** 2025-10-31

**Authors:** Moumita Dhara, Kusum Devi Vemula, Ziaul Karim, Anoop Narayanan Vadakkepushpakath, Tanvi Shetty, Anushree Prakasha Munchinamane

**Affiliations:** 1 Department of Pharmacology, Nitte College of Pharmaceutical Sciences, Nitte (Deemed to be University), Bengaluru 560064, India; 2 Department of Pharmaceutics, Nitte College of Pharmaceutical Sciences, Nitte (Deemed to be University), Bengaluru 560064, India; 3 Department of Pharmaceutics, NGSM Institute of Pharmaceutical Sciences, Nitte (Deemed to be University), Mangaluru 575018, India

**Keywords:** nanoliposome in situ gel complex, flavonoid, pectin coating, G-I stability, targeted colonic drug release, colon microbiota, anticancer

## Abstract

This study emphasizes overcoming the challenges of targeted drug delivery in colon cancer therapy by developing gastrointestinal (GI) stable, pectin-coated nanoliposomes for the oral delivery of Apigenin-Cyclodextrin Complex as an in situ gel formation. Initially, the formulation was strategically designed using design expert software for formulation optimization. FTIR and XRD studies were conducted to ensure physical compatibility and to confirm the encapsulation of apigenin within the formulation. In process optimization, among all seventeen formulations run tested, PNL (Api-Cy)-13 was identified for the highest drug loading, favourable size dimension of particle with zeta potential, and spherical external morphology through SEM analysis. The metered drug release during an in vitro study for PNL (Api-Cy)-13 was remarkably high (more than 75% of drug availability in the colonic environment, precisely in contrast to only 20% in the gastric phase in a sustained release manner), focused on colon drug targeting as an in situ gel. Furthermore, apigenin release from PNL (Api-Cy)-13 in an ex vivo chick ileum permeability study was observed both in the absence and presence of 1% vancomycin. An incremental apigenin release in the absence of the antibiotic (1% vancomycin) indicated gut microbial-associated and pectinase-mediated drug release. Here, pectin degradation materializes by the colonic microbial environment, which facilitates desirable incremental colonic drug permeation. Finally, an in vitro MTT assay and a competitive flowcytometric cell uptake study with PNL (Api-Cy)-13 using HCT-116 cells proved significant superiority in cytotoxicity profile for apigenin when delivered as an optimized coated nanoliposome in comparison to free apigenin or other non-modified nano-formulation. Also, the inhibition of the cell efflux process was validated by Multidrug Resistance 1 (MDR1) gene regulation. These observations establish an undoubted promise for the novel biopolymer, pectin-based apigenin-cyclodextrin nanoliposomes as targeted therapy in colon cancer with significant in vivo pharmacokinetics and safety profile.

## 1. Introduction

Colorectal cancer (CRC) remains a significant health challenge worldwide, accounting for approximately 1.9 million new cases and 900,000 deaths annually. By 2040, CRC incidence is predicted to increase to 3.2 million in countries with a high Human Development Index (HDI) [1]. Being the second most common cause of cancer-related death globally, CRC often requires aggressive and costly treatment approaches such as chemotherapy, radiation therapy, and hormone therapy, with a novel therapeutic approach. Also, with advancements in conventional treatments, the associated side effects and financial burden remain major concerns [2].

Even when FDA-approved immunotherapies have shown great clinical promise, their severe side effects often restrict long-term usage, further emphasizing the need for low-toxicity, naturally derived therapeutic strategies. Evidence showed that 90–95% of cancer cases are linked to environmental and lifestyle factors, underscoring the potential for prevention through naturopathy. Consequently, attention is shifting toward safer and more sustainable alternatives, such as plant-based bioactive compounds that can delay cancer progression while minimizing systemic toxicity [3].

Phytochemicals—bioactive compounds in fruits, vegetables, and grains—exhibit promising anticancer properties, modulating cellular pathways for chemoprevention and therapy. Flavonoids, anthraquinones, and tannins illustrated strong antioxidant, anti-inflammatory, and anticancer effects; the novel candidate apigenin, a flavonoid, exemplifies these properties with a broad therapeutic latent. However, the clinical claim of flavonoids is restricted due to their inherent pharmacokinetic challenges, such as low aqueous solubility, poor gastrointestinal stability, and susceptibility to rapid metabolism, leading to insufficient bioavailability [4]. Moreover, ensuring effective oral delivery with desired therapeutic levels in the colon remains a significant challenge.

Over recent decades, nanotechnology-enabled drug delivery platforms have been explored to overcome these limitations and enhance the therapeutic efficacy of phytochemicals. Innovations such as metal nanocrystals, nanohydrogels, and lipid-based carriers have demonstrated potential in improving solubility, stability, and targeted delivery. (nano-based drug delivery system). However, the use of synthetic polymers in many nanocarrier systems may pose a risk of systemic toxicity and necessitate extensive regulatory approval [5]. As a safer alternative, natural biopolymers—especially polysaccharides—have gained attraction due to their physiological compatibility, biodegradability, and capacity to form effective delivery systems. Moreover, their unique 3D structure with pH-responsive gel formation characteristics refers to proper intermolecular interaction at the cellular level to facilitate drug penetration or intercellular drug uptake and tissue drug permeation [6,7].

A promising approach to intensify apigenin delivery, involving complexation with cyclodextrins encapsulated into a nanocarrier nanoliposome, expands solubility, stability, and drug loading of apigenin [8]. Adding a methylated pectin coating in a scientifically optimized manner to nanoliposomes offers targeted oral delivery to the colon, via protection from the gastrointestinal environment [9].

The ability of methylated pectin to form in situ hydrogels in an acidic environment through hydrogen bond cross-linking makes it highly valuable in nano-formulations for colon-targeted drug delivery [10]. Upon oral administration, the pectin-coated formulation remains intact by forming a gel matrix in the acidic stomach, but in the colon, pectin degrades via pectinase due to pH changes and gut microbial fermentation. This gelation enhances drug retention till the late intestinal stage, protects active compounds like apigenin, and enables sustained, localized release at the target site through enhancing apigenin’s stability, bioavailability, and therapeutic efficacy for colon cancer treatment [11]. In this current study, a formulation design optimized for colon-localized release was demonstrated. A related mechanistic approach towards a noticeable improvement of cytotoxicity against colon cancer cells with the use of optimized pectin-coated apigenin cyclodextrin embedded nanoliposomes was also established.

The prepared formulation was developed as a colloidal suspension, intended for oral administration. Upon passage through the gastrointestinal (GI) tract, the formulation is designed to transform into a soft in situ gel, thereby providing protective encapsulation for the liposome-entrapped apigenin–cyclodextrin complex. When the formulation reaches the intestine, particularly in the colonic regions, the gel matrix will be reverted gradually to a colloidal suspension. Additionally, the colonic bacterial milieu will promote the bio-degradation of the pectin-coated nanoliposomes, leading to the controlled release of the apigenin–cyclodextrin complex. Finally, the liberated apigenin will facilitate enhanced drug absorption through increased local availability while circumventing the P-glycoprotein (P-gp) efflux mechanism, thereby ensuring optimal therapeutic efficacy.

This innovative approach shows promise for future applications in delivering further poorly soluble phytochemicals and targeted therapies, potentially transforming the oral drug delivery systems as a worthwhile option for various gastrointestinal and inflammatory diseases [12].

## 2. Results and Discussion

### 2.1. Pre-Formulation Study Using FTIR

**Figure 1:**
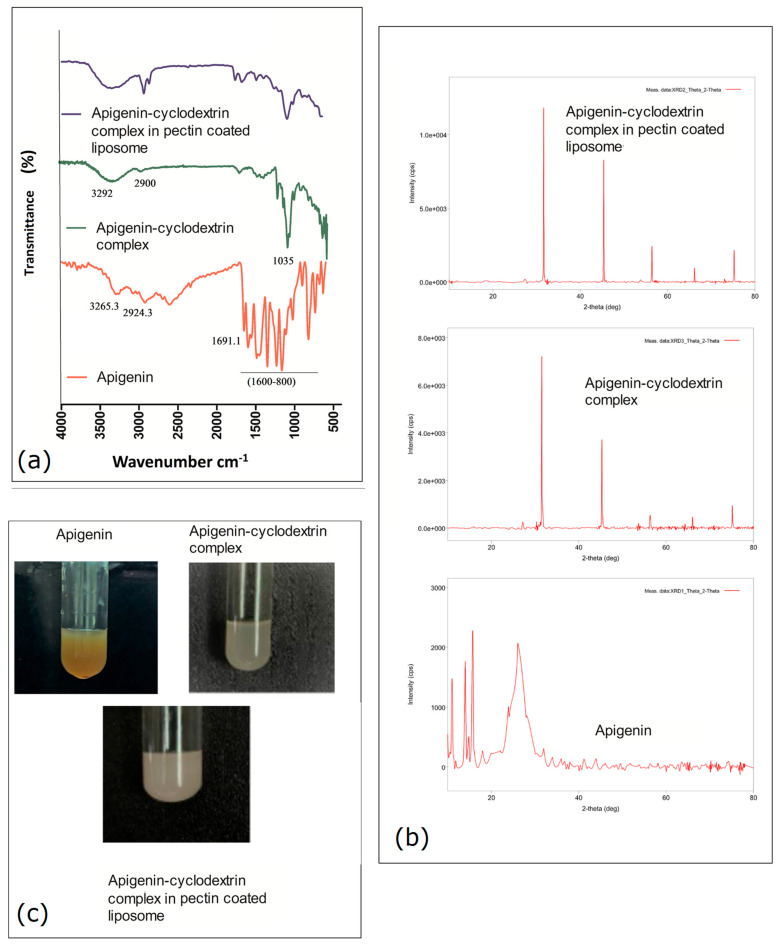
Pre-formulation study using FTIR, (**a**) and XRD, (**b**) study of apigenin, apigenin–cyclodextrin complex, and apigenin–cyclodextrin complex in pectin-coated liposome showed in (**c**).

A pre-formulation study was undertaken using Fourier Transform Infrared Spectroscopy (FTIR) to ensure the encapsulation of apigenin, as well as to evaluate the interactions between the formulation excipient and free drug apigenin. Here, the characteristic IR peak of apigenin—at 3265 cm^−1^ (O–H bond stretching), sharp stretching of (C=O bond) at 1691 cm^−1^, with signature (aromatic stretching of C=C) at 1600 to 1450 cm^−1^ range, with 1150–800 cm^−1^ (C–OH bending vibrations)—is shown in (Figure 1), where, on formation development, neither the apigenin-cyclodextrin complex nor the final (apigenin-cyclodextrin) pectinated nanoliposomes was observed to comprise the characterized IR peak for apigenin. The absence of all the characteristic peaks of apigenin indicates that apigenin is successfully encapsulated within the encapsulation process.

For a compatibility study, the FTIR of apigenin, soya lecithin, and cholesterol, as well as their combination in the form of nano-liposomal apigenin (a formulation already thoroughly observed in our previous study) was performed to assess the molecular interactions among the composition excipients [13].

The nonappearance of apigenin’s characteristic FTIR peaks when studying both the apigenin–cyclodextrin complex and the experimentally optimized pectinated nanoliposomes demonstrated the successful encapsulation of the drug and no chemical interaction with excipients [14].

### 2.2. XRD Observation Analysis

The raw apigenin exhibited multiple sharp and intense diffraction peaks, particularly at 2θ values around 11.04°, 15.8°, 26.5°, 27.1°, and 31.2°, indicating its highly crystalline nature.

In contrast, the pectin-coated apigenin-loaded nanoliposome powder (PNL (Api-Cy)-13) showed a noteworthy decrease in intensity and in a series of characteristic apigenin peaks. The XRD pattern of the nanoliposome formulation presented only one or two sharp peaks, which may be attributed to the pectin coating. This result suggested a partial or complete amorphization of apigenin upon encapsulation within the liposomal matrix and subsequent coating with pectin. This transformation can be attributed to the molecular dispersion of apigenin in the lipid bilayer and possible interactions with pectin.

The disappearance of numerous sharp XRD peaks of crude apigenin following its encapsulation with pectin-coating on the nanoliposomes suggests distinct amorphization [15].

### 2.3. Formulation Optimization Strategy by Design Expert

After the analysis of all the formulation (designed as described in Table 1 and Table 2, following central composite design) characterization responses (Table 3), except for the in vitro drug release in intestine simulated pH in the presence of pectinase, the Design Expert software proposed a quadratic model, however a linear model was recommended for in vitro drug release in intestine simulated pH in presence of pectinase. Based on the ANOVA through model F value and *p*-value, the model terms A/B/C/AB/AC/BC/A^2^/B^2^/C^2^ were identified as significant. The observed non-significant lack of fit was good for the model, which was optimized to remain within the intended design limits (Table 1). Quadratic/linear relations were produced by the Design Expert software, based on the responses obtained (Table 3) for the prepared liposomes (F1–F17). Equations (2)–(7) for all the responses are given below:

Drug Loading

Y_1_= 90.38 + 21.74A − 15.34B + 0.20C + 20.30AB + 0.25AC + 0.50BC − 5.66A^2^ − 9.66B^2^ − 8.16C^2^. (1)

Particle Size

Y_2_ = 319.97 + 21.20A + 15.50B + 251.20C − 13.38AB + 34.62AC − 22.13BC − 4.45A^2^ + 15.05B^2^ + 106.55C^2^. (2)

Zeta Potential

Y_3_ = −7.47 + 1.63A + 0.0578B − 12.39C−1.10AB + 2.08AC + 0.379BC − 1.19A^2^ + 0.4575B^2^ − 4.17C^2^. (3)

In vitro drug release in gastric simulated pH at 4 h

Y_4_ = 22.87 − 0.90A + 2.80B − 19.30C − 1.75AB + 1.25AC − 2.25BC − 2.53A^2^ + 2.97B^2^ + 7.47C^2^. (4)

In vitro drug release in intestine simulated pH in absence of pectinase between 4 and 8 h

Y_5_ = 52.97 + 2.90A − 2.60B + 7.60C + 1.25AB−0.25AC + 1.25BC + 9.05A^2^ − 4.45B^2^ − 10.45C^2^. (5)

In vitro drug release in intestine simulated pH in presence of pectinase

Y_6_ = 77.12 + 2.10A + 0.10B − 11.80C (6)

The statistical equations derived using Design Expert software describe how the formulation variables A, B, and C influence six key responses of liposomal formulations (F1–F17), with most responses fitting a quadratic model except for drug release in intestinal pH with pectinase, which followed a simpler linear model. The quadratic models—supported by significant ANOVA model F-values and low *p*-values for terms A, B, C, their interactions (AB, AC, BC), and quadratic terms (A^2^, B^2^, C^2^)—indicate strong and nonlinear relationships between formulation variables and the outcomes. A non-significant lack of fit validates the model’s applicability within the design space. Equation (2) shows that drug loading (Y_1_) is positively influenced by A and the AB interaction but negatively impacted by B, A^2^, B^2^, and C^2^, suggesting that higher levels of A enhance loading, while excessive B or high curvature in factor levels reduces it. Equation (3), for particle size (Y_2_), highlights that factor C has the most dominant positive effect, indicating that increases in C lead to significantly larger particles; the interaction terms and quadratic effects further refine this outcome, suggesting careful control of C is needed to avoid oversized particles. Equation (4) for zeta potential (Y_3_) reveals a negative baseline value, with C having a strong inverse effect, meaning higher C reduces stability; A and B have minimal positive contributions, while AC and C^2^ are also influential, showing the importance of maintaining C at an optimal range for stability. In Equation (5), which models drug release in gastric pH (Y_4_), B enhances drug release, while C drastically reduces it, and the quadratic term C^2^ further emphasizes this suppression, indicating that higher surfactant or lipid levels may slow gastric release, beneficial for targeting intestinal delivery. In Equation (6), the intestinal release in the absence of pectinase (Y_5_) is positively impacted by A and C, while B shows a negative influence; again, A^2^ and C^2^ are important, indicating that optimal, balanced levels of these factors promote sustained release over 4–8 h. Finally, Equation (7) for Y_6_ shows a simple linear model was sufficient when pectinase was present, with A slightly increasing and C significantly decreasing drug release, indicating that in enzyme-rich conditions, C is a dominant suppressor, perhaps due to interactions with the enzyme or formulation matrix. The simplicity of this model implies a more straightforward mechanistic pathway under these enzymatic conditions. Collectively, these models demonstrate the robust utility of statistical tools in optimizing liposome formulations by identifying critical factor effects and interactions, allowing for precise tuning of formulation parameters to achieve desired outcomes like high drug loading, appropriate particle size, stable zeta potential, and controlled release behaviour across gastrointestinal conditions [16]. The model fit summary suggests the type of model (Quadratic/Linear); R^2^ determines the correlation between predicted and observed values.

Formulations (F1–F17) were optimized using a central composite design (CCD) to evaluate the influence of formulation variables on particle size, zeta potential, drug loading, and in vitro release. ANOVA (Table 4a,b) confirmed the adequacy of quadratic models with high significance and minimal lack-of-fit. Drug release studies demonstrated limited release at gastric pH, sustained release in intestinal pH without pectinase, and enzyme-triggered accelerated release in the presence of pectinase. Regression analysis (Supplementary Table S1) further validated the predictive strength of the models. Overall, CCD-based optimization established a robust, reproducible system with controlled, site-specific release, highlighting its potential for targeted drug delivery applications.

### 2.4. Physical Evaluation of Optimized Formula

#### 2.4.1. Drug Loading

The drug loading capacity of the experimental nanoformulations for the optimized cyclodextrin–apigenin complex and coated cyclodextrin-apigenin complex nanoliposomes (showed in design optimization table) comprised 90–93% of drug loading where plain nanoliposomal apigenin without a cyclodextrin–apigenin complex demonstrated about 80–85% drug loading efficiency.

The elevated drug loading was observed for the cyclodextrin–apigenin complex nanoliposomes PNL (Api-Cy)-13 in comparison to plain apigenin liposomes. Correspondingly, apigenin-loaded lipid nanocapsules established encapsulation efficiencies of ~95.9% [17].

#### 2.4.2. Particle Morphology Characteristics

**Figure 2:**
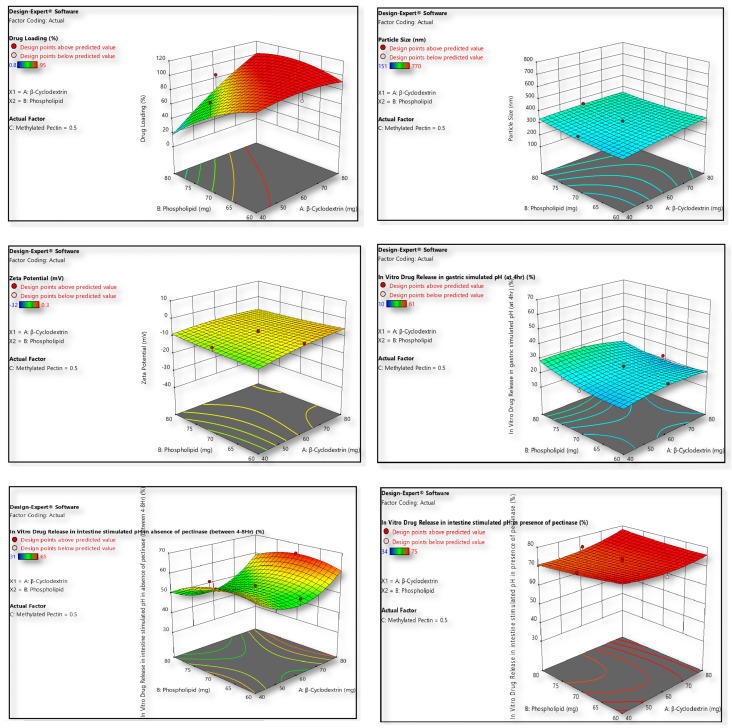
Pictorial representation of formulation optimization obtained by Design Expert software.

The design optimization table also depicted that the optimized nanoliposomes PNL (Api-Cy)-13 formulation exhibited nano-sized characteristics (~ size 350 nm) with a zeta potential of −25.1 mV for experimental pectin-coated cyclodextrin–apigenin complex nanoliposomes. Finally, the surface morphology was confirmed using field emission electron microscopy (FESEM) as depicted in (Figure 2), where homogenized dispersed optimized nanoliposomes was also observed with 350 nm range.

#### 2.4.3. Swelling Index (SI)

The observed swelling index of the lyophilized pectin-coated nanoliposome formulation (Api-Cy-13) demonstrates a significant pH-dependent behaviour, depicted in Supplementary Figure S1. Specifically, in simulated gastric fluid (SGF, pH 1.2), the formulation exhibits a swelling index of approximately **90%**, indicating substantial gel formation under acidic conditions. In contrast, in simulated intestinal fluid (SIF, pH 6.8), the swelling index decreases to less than **20%**, reflecting a transition to a more colloidal state. This pH-responsive swelling behaviour is characteristic of pectin-based systems, where the protonation of carboxyl groups at a low pH leads to gelation, while ionization at a higher pH results in disintegration.

Such a profile is advantageous for colon-targeted drug delivery, ensuring that the formulation remains intact, protecting the encapsulated apigenin in the acidic stomach environment, and releasing the drug in the higher pH of the colon. In regard to pectin-based delivery systems with nanoconjugates, this present study highlights their potential for controlled and site-specific drug release [18].

The experimental observation in optimized nanoliposomes PNL (Api-Cy)-13, indicating colloidal stability and successful surface modification, shows that pectin adsorption on liposomal surfaces increases particle size and shifts zeta potential to negative values. FESEM images confirmed this with uniformly dispersed, spherical nanovesicles around this size [19].

### 2.5. In Vitro Drug Release Pattern Applying GI Simulated Media

**Figure 3:**
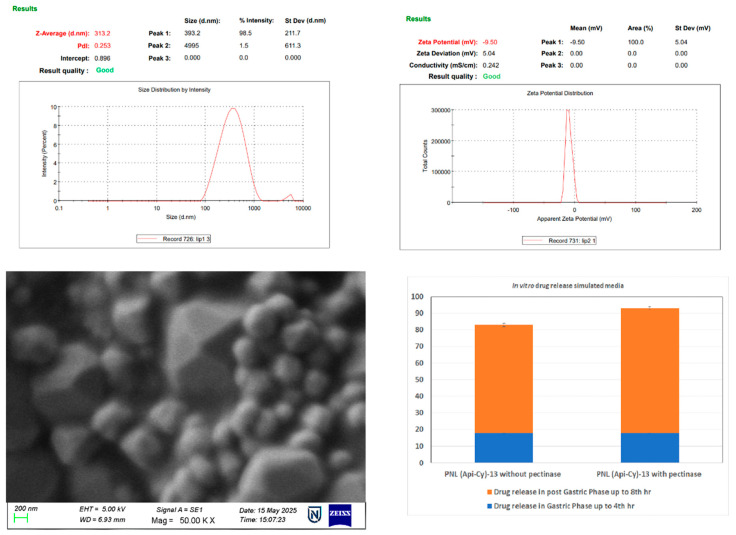
Physiochemical characterization profile for optimized nanoparticles.

The in vitro drug release study employed a biorelevant digestion model based on the standardized INFOGEST protocol to simulate human gastrointestinal conditions. Seventeen apigenin-loaded nanoliposome formulations, both coated and uncoated, were evaluated using a dialysis method in simulated gastric fluid (SGF) and simulated intestinal fluid (SIF). During the gastric phase (pH 3.0), pectin-coated formulations showed significantly reduced drug release (10–20%) compared to uncoated formulations (Figure 3) which released approximately 40% of the drug. This highlights the gastric protective effect of the methylated pectin coating. Upon transitioning to the intestinal phase (pH 6.2), drug release from the coated formulations increased markedly, particularly in the presence of 3% pectinase, simulating microbial activity in the colon.

The pectin coating facilitated in situ hydrogel formation in the acidic environment via hydrogen bonding, which maintained formulation integrity in the upper GI tract. In the colon, the enzymatic degradation of pectin by pectinase allowed for the controlled and targeted release of apigenin. This behaviour supports the potential of methylated pectin as a colon-specific delivery vehicle [20].

The in vitro release findings align with previous reports by Gutiérrez-Alvarado et al. [21], where pectin-based hybrid microspheres exhibited pH-dependent, colon-targeted release due to controlled swelling and enzymatic degradation. Similarly, the current INFOGEST-based study confirmed that methylated pectin coating suppressed gastric apigenin release (10–20%) while promoting enzyme-triggered colonic delivery, validating its efficiency as a colon-specific carrier.

### 2.6. Ex Vivo Intestinal Drug Release in Presence and Absence of Vancomycin

**Figure 4:**
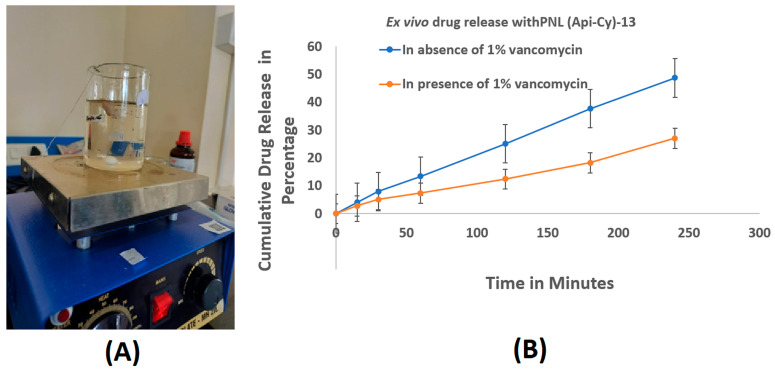
Ex vivo drug release study: (**A**) study arrangement; (**B**) estimation of release profile for the conducted study.

The ex vivo intestinal permeability study using chicken ileum sacs demonstrated a significant difference in the drug release profile of the optimized apigenin nanoformulation in the presence and absence of 1% vancomycin. In the absence of vancomycin, a sustained and higher release of apigenin was observed (Figure 4), indicating effective degradation of the pectin coating by endogenous microbial enzymes, particularly pectinase. This enzymatic action is crucial for triggering the breakdown of the pectin matrix, thereby facilitating the release and permeation of apigenin across the intestinal wall. Conversely, the formulation exposed to the vancomycin-applied intestinal sac demonstrated a markedly lower drug release throughout the 120 min study period. This can be attributed to vancomycin’s broad-spectrum antibacterial activity, which disrupts the gut microbial flora responsible for producing pectinase. Without adequate pectinase activity, the pectin coating on the nanoliposomes remains largely intact, limiting drug diffusion through the intestinal membrane. These findings suggest a microbiota-dependent release mechanism, where bacterial enzymes play a pivotal role in degrading the pectin layer to enable apigenin release.

The study highlights the importance of local microbiota in modulating drug release from pectin-based delivery systems. The observed reduction in apigenin release in the presence of vancomycin confirms the dependency of the formulation on bacterial enzyme-triggered degradation. This underscores the formulation’s potential for colon-targeted delivery, leveraging microbial enzyme activity for site-specific drug release, which may be compromised during antibiotic therapy [22]. Ex vivo studies revealed that vancomycin markedly inhibited apigenin release by suppressing pectinase-producing microbiota, confirming a microbiota-dependent degradation of the pectin coating and its critical role in colon-targeted release.

### 2.7. Cytotoxicity Profile of Experimental Coated Nanoliposomes

**Figure 5:**
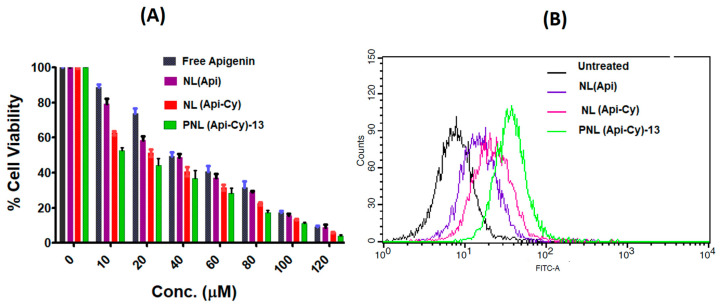
Cell cytotoxicity (**A**) and FACS cell uptake study (**B**) analysis using optimized pectinated apigenin cyclodextrin nanoliposomes in comparison to free apigenin and plain apigenin nanoliposomes.

The MTT assay results demonstrated that the modified pectin-coated liposomes encapsulating the apigenin–β-cyclodextrin complex exhibited significantly higher cytotoxicity against colon cancer cell lines compared to plain liposomes and free apigenin, depicted in Figure 5A. The enhanced cytotoxic response is attributed to the synergistic effect of multiple formulation features. The pectin coating not only contributes to improved cellular uptake due to its mucoadhesive properties and potential affinity toward colon cancer cells but also provides stability in the gastrointestinal environment, ensuring greater drug availability at the target site.

Furthermore, the inclusion of apigenin within β-cyclodextrin enhances its aqueous solubility and protects it from premature degradation, thereby improving drug loading efficiency and sustained intracellular delivery. The nano liposomal structure facilitates efficient endocytosis and intracellular accumulation, while the pectin matrix may trigger selective interaction with cancer cells, enhancing therapeutic efficacy [23].

Overall, the superior cytotoxic profile of the pectin-coated nanoliposomes can be linked to improved pharmacokinetic behaviour, controlled drug release, and enhanced cellular permeability. These results support the formulation’s potential as a targeted oral nanocarrier for effective colon cancer therapy by maximizing intracellular delivery of apigenin and reducing off-target effects.

### 2.8. Cellular Uptake Study Using FITC-Labelled PNL (Api-Cy)-13

The flow cytometry-based cellular uptake study, Figure 5B, revealed a significant difference in the intracellular accumulation of FITC-labelled formulations in HCT116 cells. The optimized pectin-coated nano-cyclodextrin–apigenin formulation exhibited markedly higher fluorescence intensity compared to the plain apigenin nano formulation, indicating enhanced cellular uptake. This enhanced uptake can be attributed to the presence of the pectin coating, which facilitates cohesion and prolonged interaction with the cell membrane, along with cyclodextrin-mediated solubility enhancement and protection of the apigenin payload.

Flow cytometry showed significantly higher FITC uptake in HCT116 cells treated with pectin-coated nano-cyclodextrin–apigenin compared to plain liposomes, owing to pectin’s mucoadhesive properties and cyclodextrin-enhanced solubility. This aligns with evidence that polysaccharide-coated liposomes exhibit increased cellular uptake over conventional formulations [17].

Overall, the results validate the potential of the optimized nanoformulation for enhanced intracellular delivery of apigenin, improved drug accumulation in colorectal cancer cells, and possible modulation of drug resistance mechanisms through P-gp inhibition [24].

### 2.9. MDR1 mRNA Expression Analysis via RT-PCR

To substantiate the proposed mechanism of P-gp modulation suggested in Section 2.7 and Section 2.8, an **RT-PCR assay targeting the MDR1 gene (encoding P-gp)** was performed to quantify transcriptional changes following treatment with apigenin–cyclodextrin-loaded pectin nanoliposomes [25]. A reduction in MDR1 expression in the pectin-coated formulation (Api-Cy-13) was observed thoroughly using HCT-116 both in the presence (pretreated) and absence of 20 ng/mL verapamil.

**Figure 6:**
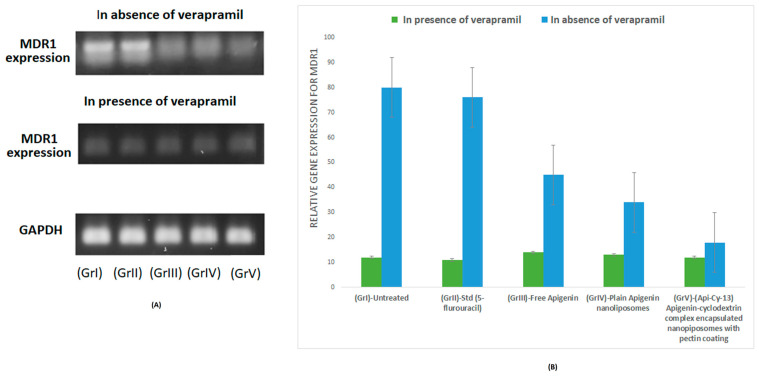
RT-PCR study; (**A**,**B**) MDR1 comparative expression level.

As verapamil is a P-gp inhibitor, in the presence of verapamil, MDR1 expression is very low across all treatment groups. However, a different MDR1 expression level was observed; (Api-Cy-13), depicted in (Figure 6), demonstrated the lowest MDR1 expression when prior verapamil treatment was not executed. Compared to free apigenin-treated, along with plain apigenin and encapsulated nanoliposomes formulations, the treated cells confirmed the transcriptional downregulation of P-gp, thereby supporting enhanced intracellular accumulation observed in flow cytometry assays.

### 2.10. In Vivo Studies

#### 2.10.1. Intestinal Pharmacokinetics

**Figure 7:**
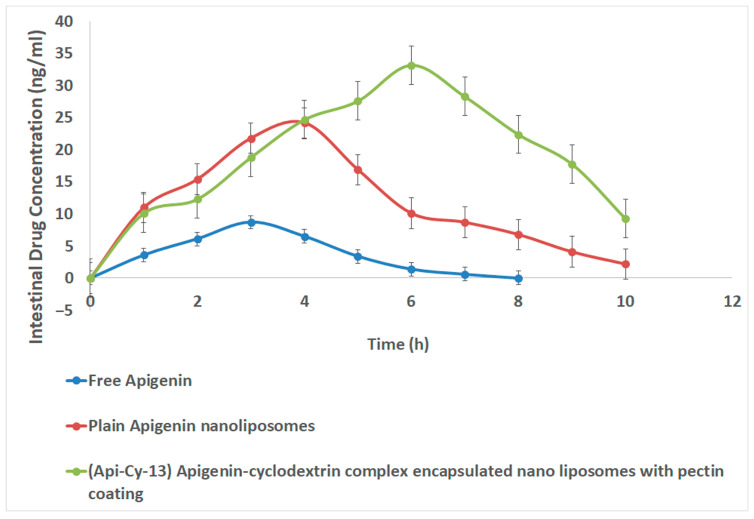
In vivo intestinal pharmacokinetics apigenin concentration study.

The (Api-Cy-13) formulation demonstrated (Figure 7; Table 5) remarkable improvement in the pharmacokinetic performance of apigenin compared to the free drug and plain nanoliposomes. Specifically, Cmax increased by approximately 3.8-fold, and AUC_0_–last increased by 6.6-fold relative to free apigenin, clearly indicating enhanced absorption and systemic bioavailability. The significant rise in Tmax and mean residence time (MRT) further suggests a controlled and sustained release of apigenin from the pectin-coated nanoliposomes, effectively prolonging its circulation time in plasma.

Additionally, the observed reduction in clearance reflects a slower elimination rate, likely due to the protective effects of the pectin coating and cyclodextrin complexation, which may reduce enzymatic degradation and improve the metabolic stability of apigenin. The combined effects of cyclodextrin inclusion, enhancing solubility and permeability, and pectin-based coating, providing mucoadhesive and sustained-release characteristics, contributed synergistically to the improved pharmacokinetic profile.

Overall, these findings indicate that the (Api-Cy-13) system successfully enhances the solubility, stability, intestinal retention, and oral bioavailability of apigenin, demonstrating its potential as an effective delivery platform for poorly water-soluble bioflavonoids [26].

#### 2.10.2. Systemic Safety Studies

**Figure 8:**
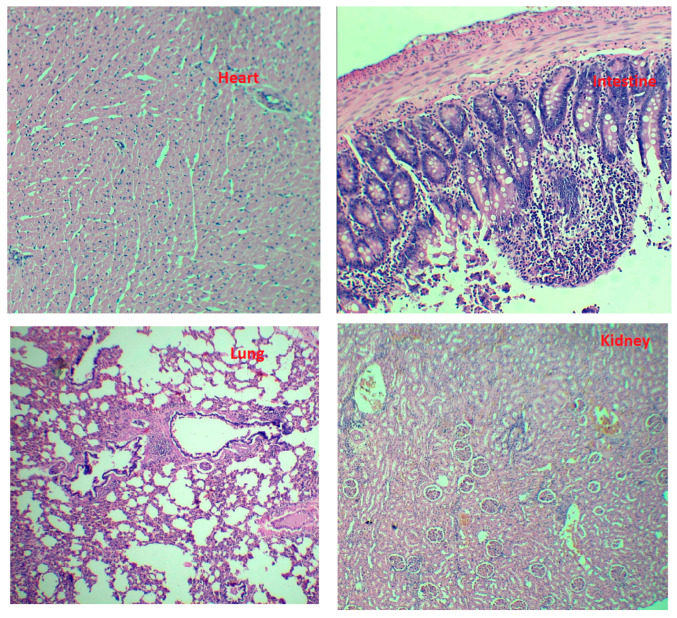
Histopathological examination on (Api-Cy-13) oral administration.

Histopathological evaluation of major organs (Figure 8), including the liver, kidney, heart, and intestine, revealed no significant structural or cellular abnormalities, indicating the absence of overt toxicity. This observation confirms the biocompatibility of the (Api-Cy-13) apigenin–cyclodextrin complex encapsulated within pectin-coated nanoliposomes, with the lack of toxicity likely attributable to the protective encapsulation that mitigates direct tissue exposure during systemic drug administration

## 3. Conclusions

This work demonstrates the successful engineering of a methylated pectin-coated β-cyclodextrin–apigenin nanoliposomal platform, offering a microbiota-responsive and colon-specific oral delivery strategy for poorly soluble phytochemicals. The optimized system exhibited high drug loading, nanoscale dimensions, colloidal stability, and enzymatically triggered release, resulting in markedly enhanced cytotoxicity and cellular uptake over free apigenin and uncoated liposomes. Vancomycin-mediated suppression of release confirmed gut microbial enzymes as critical modulators, while P-gp inhibition underscored its potential to circumvent efflux-mediated drug resistance. These findings position the formulation as a versatile nanocarrier blueprint for oral delivery of bioactive plant-derived molecules, capable of improving bioavailability, site-specific targeting, and therapeutic efficacy. Future investigations should integrate in vivo pharmacokinetic and pharmacodynamic profiling, microbiome–drug interaction mapping, and long-term safety assessments to accelerate translation into clinical phytopharmaceutical applications.

## 4. Materials and Methods

### 4.1. Materials and Instruments

Apigenin (AP) with 97.95% purity was purchased from sigma, India. Soy lecithin (>94% phosphatidylcholine), cholesterol (≥99%), beta cyclodextrin (purity), pectin (low molecular weight, ≥75% degree of deacetylation), pepsin (porcine, ≥500 U/mg), pancreatin (for porcine, 4× USP) and porcine bile extract were obtained from Sigma-Aldrich (St. Louis, MO, USA). Acetone, acetonitrile, methanol (HPLC grade), hydrochloric acid, sodium chloride, sodium carbonate, and all chemicals used were of analytical grade unless specified. Water deionized from Milli-Q system (Millipore, Bedford, MA, USA) was used for all analyses.

Cell lines: colorectal cell lines HCT116 were previously purchased from NCCS, Pune, and maintained.

#### 4.1.1. Pre-Formulation Studies

##### FTIR Study

FTIR analysis was conducted using a Bruker ATR Alpha at 25.0 ± 0.5 °C. The procedure was a direct sample method, without palette formation. A few milligrams of the sample were placed on the zinc selenide, and spectra were scanned from 4000 to 500 cm^−1^ to assess functional characterization groups, drug-excipient compatibility, and proper drug entrapment [27].

##### XRD Analysis Study

To assess the crystallinity and phase behaviour of the samples, an X-ray diffraction (XRD) study was conducted on the raw drug Apigenin, the freeze-dried pectin-coated apigenin-encapsulated nanoliposome powder (PNL (Api-Cy)-13), and the blank nanoliposome formulation (PNL (Api-Cy)-13 without Apigenin). Each sample was placed on the sample receptacle and gently compressed to ensure a flat and even surface, minimizing preferred orientation effects during analysis.

The diffraction forms were chronicled using a Shimadzu XRD-7000 X-ray diffractometer (Shimadzu, Kyoto, Japan). The instrument was operated in 2θ scanning mode across a range of 5° to 90°, with a step size of 0.02° and a counting time of 1 s per step. The X-ray source utilized Cu Kα radiation (λ = 1.5406 Å), operating at 40 kV and 30 mA [28].

#### 4.1.2. Design of Experiments (DOE) Strategy for Formulation Optimization

The present study constructs quadratic/linear equation through the CCD (central composite design) model to estimate the response variables employing three-level factorial trials, operated by Design Expert (Version 11, State-Ease, Inc., Arden Hills, MN, USA.) [29]. Here, the optimization method was eased with 17 tentative runs. In current formulation β-cyclodextrin (A), phospholipid (B), and methylated pectin (C) were chosen as independent variables while considerable dependent variables included were drug loading (Y_1_), particle size (Y_2_), zeta potential (Y_3_), in vitro drug release in gastric simulated pH at 4 h (Y_4_), in vitro drug release in intestine simulated pH in absence of pectinase between 4 and 8 h (Y_5_) and in vitro drug release in intestine simulated pH in presence of pectinase (Y_6_). Succeeding the designed trials, the independent variables were assessed as low level (−1), medium (0), and high level (+1). A design background was set up, encompassing 17 experimental runs, including three replications at the centre point to estimate pure error for the lack of fit test. The general second-order polynomial regression model obtained is represented below as Equation (1).

Y = b_0_ + b_1_A + b_2_B + b_3_C + b_4_AB + b5AC + b6BC + b7A^2^ + b8 B^2^ + b9C^2^ (7)

In this equation, Y denotes the response variable or the dependent variable whose value is influenced by changes in the independent variables. The variables A, B, and C are the independent variables or factors being studied. The coefficient b0 is the intercept or constant term, representing the value of Y when all independent variables are equal to zero. The coefficients b_1_, b_2_, and b_3_ represent the effects of the linear terms of the variables A, B, and C, respectively. These indicate how each factor individually influences the response. The coefficients b_4_, b_5_, and b_6_ correspond to the two-way interaction terms AB, AC, and BC, respectively. The relationships capture the combined effect of two variables acting together on the response, which may differ from the sum of their individual effects. Finally, the coefficients b_7_, b_8_, and b_9_ are associated with the quadratic terms A^2^, B^2^, and C^2^. These terms allow the model to capture curvature in the response surface, making it possible to identify nonlinear relationships and optimize the response more effectively.

#### 4.1.3. Preparation of Cyclodextrin-Embedded Coated Nanoliposomes

##### Preparation of Apigenin and β-Cyclodextrin Inclusion Complexes Using Ani-Solvent Solution

For the preparation of apigenin and β-cyclodextrin inclusion complexes, apigenin (10 mg) was dissolved in a 1 mL solvent (acetone: dimethyl sulfoxide ~ 1:1) and then the prepared apigenin solution was injected dropwise into a 5 mL of β-cyclodextrin solution, which was then placed on magnetic stirrer rotating at 100 rpm. We maintained the molar ratio of apigenin: β-cyclodextrin~1:1 to 1:2 (40 to 80 mg of β-cyclodextrin to maintain the molar ratio with drug apigenin) to achieve maximum drug loading in the final optimized formulation. We placed this arrangement on a magnetic stirrer at (40–50) °C till to precipitate the residue of apigenin- β-cyclodextrin inclusion complexes [30,31].

##### Preparation of Nanoliposomes

During the preparation of nanoliposomes, the prepared solvent residue apigenin-β-CD complex, 60–80 mg of soya lecithin, and one third amount of cholesterol of lipid (as per the previously developed protocol of nanoliposome preparation in the lab) was combined with 5 mL of chloroform in a round-bottom flask and subjected to rotary evaporation to remove the organic solvent and to cast a thin film on the round bottom surface. Any residual solvent also evaporated as the sample was then placed in the desiccator to completely dry. Here, we followed the previously established central composite design to optimize the formula (described in Table 1 and Table 2). The next day, the properly dried thin film was dissolved in an aqueous solution comprising phosphate buffer (pH 7.4) and sonicated to produce a uniform lipid solution. Then, the lipid solution was kept in resting phase overnight to form uni-layer nanoliposomes. The nanorange particles were separated through centrifuge and finally lyophilized for further storage [13].

##### Coating of Pectin on Liposomes and Its Purification

The pectin-coated liposomes were prepared by adding 1 mL liposomal dispersion to 4 mL pectin solution drop wise (∼1.2–5.5 mL/min) under continuous magnetic stirring with such a speed that a swirl could be seen in the pectin solution. Here, we also followed the previously established central composite design (pectin 0.5% to 1% of pectin for optimizing the formula as described in Table 1 and Table 2). The coated liposomal dispersion was then transferred to polycarbonate centrifuge tubes and ultra-centrifugated (RAMI C24 plus, Mumbai, India) at 3000 rpm to separate coated nanoliposomes and form a dispersion following the size distribution of experimental nanoliposomes [19].

#### 4.1.4. Physicochemical Characterization (In Vitro)

##### Drug Entrapment

In drug loading for the flavonoid (apigenin), we correctly weighed (2 mg) of the different experimental samples as performed via strategic experimental design (according to Design Expert and following central composite design). Each were dissolved in a mixture solution of ethanol-acetonitrile-dimethyl sulfoxide (2 mL solvent, at a ratio of 0.5:1:1 (*v*/*v*) as the best compatible solvent composition for estimating drug loading in the experimental formulations. The final solution was mixed for 1–2 h and then centrifuged for 20 min at 10,000 rpm. The obtained indistinct and opaque supernatant (1 mL) was separated to measure the absorbance intensity using the UV/VIS-spectrometer at the corresponding λ_max_ of apigenin, 234 nm. The drug content in the test liposomes was analyzed with the corresponding standard calibration curve prepared previously using various samples of a serial concentration, prepared as follows: 5 µg/mL, 10 µg/mL, 15 µg/mL, 20 µg/mL, 25 µg/mL. Accordingly, the percentage of drug loading and encapsulation efficiency were calculated using the following equations [32].

➢ % Drug loading = (amount of drug in liposome/amount of liposome used) × 100
➢ % Yield = (weight of dry powdered liposome/total weight of all the components used in the formulation) × 100

##### Particle Size and ζ- Potential Extents

The particle size distribution and ζ-potential of the experimental formulation differed from the one performed via strategic experimental design (according to Design Expert and following central composite design) were analyzed using a Malvern Zetasizer Instrument (Nano-ZS 90, Worcestershire, UK) applying a dynamic light scattering (DLS) technique with the sample suspension of the experimental liposomes by diluting it properly through vortex and sonication process. The results represented were the average particle size, considering the standard deviation of at least three different batches of the experimental liposomes [33].

##### SEM Study for Particle Morphology Analysis

Lyophilized experimental formulations were suspended on the surface of double-sided carbon tape coated with a thin layer of gold. The morphology of elements was observed with a Hitachi SU8010 FE-SEM (Hitachi, Co., Tokyo, Japan) operated at a voltage of 8 kV [34].

##### Determination of Swelling Index (SI)

The accurate weight of 100 mg of the lyophilized form of colloidal suspension for PNL (Api-Cy)-13 was taken in a Petri dish and allowed to swell in simulated gastric fluid (SGF, pH 1.2) for up to 4 h, again the same was performed in simulated intestinal fluid (SIF, pH 6.8), maintaining the temperature at 37 ± 0.5 °C. The swelling index was measured using the following formula.

(8) $$\mathrm{SI}=\frac{Wt.-W_{0}}{W_{0}}$$

where Wt. indicates the weight of the content after swelling, and W_0_ indicates the weight of the content before swelling.

##### In Vitro Drug Digestion and Release Study

In vitro digestion was carried out using simulated gastric fluid (SGF) and simulated intestinal fluid (SIF), based on the INFOGEST protocol [35,36].

Initially, we weighed 5 mg of the experimental formulations in a dialysis sac—here, we analyzed 17 formulations, fabricated and developed following the strategic experimental design according to Design Expert, as depicted in Table 1. The sac was then immersed in a 250 mL of beaker, placing it on a magnetic stirrer with temperature of 37 °C. Initially, the pH was adjusted to 3.0 by adding 1 M HCl and 0.5 mL of pepsin (3100 U/mg) to maintain the gastric phase. Afterwards, 5 ml of the sample was withdrawn from the beaker at time intervals of 15, 30, 45, 60, 90, 120, 150, 180, 210, and 240 min, replacing the 5 ml of buffer in the beaker each time. At each time interval, free apigenin concentration was estimated using the UV spectrometric method, referring to the previously prepared standard concentration curve of apigenin.

After 4 h, pepsin was inactivated by adjusting the pH to 6.2 using 1 M NaOH with 1 mL of pancreatin (46.78 mg/mL in SIF, 342 U/mg based on lipase) and 2.5 mL of bile salt (28.789 mg/mL in SIF, 5.56 mmol of bile salts/g bile) to establish the intestinal environment. At this time (4th h), again, 5 mL of the sample was withdrawn up for another 4 h at 30 min intervals to evaluate post gastric phase drug release following the two processes. For the first process, the release study was conducted without adding pectinase to the release media. And during second, the same simultaneous intestinal simulated drug release was performed, but in presence of 3% of pectinase to observe the late intestinal or colonic drug release profile, especially resembling the colonic bacterial environment. Here, free apigenin concentration was also estimated using observed absorbance by UV spectrometric method [18].

Finally, a cumulative drug release profile was calculated to understand the digestion of the orally delivered apigenin, encapsulated in the experimentally designed coated nanoliposomes formulation.

#### 4.1.5. Evaluating Intestinal Drug Release Profile of Optimized Nanoformulation in Presence of 1% Vancomycin Using Chick Ileum Ex Vivo Method

An ex vivo permeability study was conducted by using chick ileum sacs. Chicken gastrointestinal parts were collected from a local butcher shop and the large intestinal part was extracted and partially washed with distilled water to eliminate the excess mucous. Thereafter, unwanted fluid from the gastrointestinal lumen was removed and deposited in an oxygen chamber. The 3 cm long isolated intestinal lumen sacs were used as a drug pocket by tying up the two ends of the sac. Here, the formulation PNL (Api-Cy)-13, optimized by strategic design, was used to analyze the drug release pattern of apigenin both in the presence and absence of 1% vancomycin. The sacs were immersed into a dissolution beaker containing 250 mL of buffer (acetate buffer 6.3 around), maintained at 37 ± 0.5 °C and stirring at 50–100 rpm. Samples were withdrawn from the beaker at predetermined time intervals of 0, 15, 30, 60, 90,120,180, and 240 min. At the specified intervals, 5 mL samples were collected using a calibrated plastic syringe, and the same volume of fresh medium was introduced to restore the solution to the initial level. The samples were analyzed by the UV spectrometric method with proper dilution. The permeability of apigenin through the intestinal wall was checked at ex vivo intestinal atmosphere [37].

#### 4.1.6. In Vitro Cytotoxicity Assay and Cell Uptake Assay Using Colon Cancer Cell Lines

The in vitro cytotoxic potential for the developed optimized formulation, PNL (Api-Cy)-13, was assessed on human colorectal adenocarcinoma (HCT-116) cell lines using the MTT assay method, comparing free apigenin and non-coated plain nanoliposomes. The cells were prepared in a 96-well plates system at a confluency of 1 × 10^4^ cells/well in 100 µL DMEM accompanied with 10% FBS and penicillin–streptomycin, then incubated at 37 °C in 5% CO_2_ for 24 h. Post-incubation, the cells were treated with various concentrations of the test formulation ranging from 10 to 120 µM equivalent concentrations of apigenin [38].

After 24 h of treatment, 20 µL of the MTT solution (5 mg/mL in PBS) was added and incubated for 4 h. Viable cells converted MTT into insoluble purple formazan crystals. Post-incubation, the medium was discarded and replaced with 100 µL of DMSO to dissolve the crystals, followed by measurement of absorbance at 570 nm on a microplate reader.

#### 4.1.7. Cellular Uptake Study Using Flow Cytometry in HCT116 Cells

The cellular uptake of FITC-labelled optimized pectin-coated cyclodextrin–apigenin nanoformulation FITC- PNL (Api-Cy)-13 versus plain apigenin nanoformulation was evaluated using HCT116 colorectal cancer cell lines. Cells were seeded in 6-well plates at a density of 2 × 10^5^ cells/well and incubated for 24 h in DMEM, supplemented with 10% FBS and 1% penicillin-streptomycin at 37 °C and 5% CO_2_.

Following this, cells were treated with either FITC-labelled plain apigenin nanoformulation, apigenin–cyclodextrin complex nanoformulation, or FITC-labelled optimized pectin-coated formulation at concentrations near their respective IC_50_ values (as determined from the MTT assay). After 2 h of incubation, cells were washed with PBS, trypsinized, and collected for flow cytometric analysis [39].

The fluorescence intensity of intracellular FITC was quantified using a flow cytometer to determine the extent of cellular uptake. Comparative analysis between the two formulations was performed to assess the enhancement in intracellular drug accumulation. Data were analyzed to correlate uptake efficiency with cytotoxic potential, referencing IC_50_ values for both formulations.

#### 4.1.8. Real-Time PCR Study

Total RNA was extracted from cell lysates (of HCT-116) [40] using the TRIzol reagent (Thermo Fisher Scientific, Waltham, MA, USA) following the manufacturer’s protocol. Complementary DNA (cDNA) was synthesized from 1 µg of RNA using the Maxima First Strand cDNA Synthesis Kit (Thermo Fisher Scientific, USA). Quantitative PCR was performed using the QuantiTect SYBR Green PCR Kit (QIAGEN, Hilden, Germany) with 5 pmol of forward (5′-GAG GAG GAG GAG GAG GAG GAG G-3′) and reverse (5′-GAG GAG GAG GAG GAG GAG GAG G-3′) primers specific to the MDR1 gene [25]. GAPDH was used as an endogenous control. PCR amplification was conducted in a StepOnePlus Real-Time PCR System (Applied Biosystems, Norwalk, CT, USA) with the following cycling conditions: initial denaturation at 95 °C for 2 min, followed by 40 cycles of 95 °C for 15 s and 60 °C for 1 min. Relative gene expression was calculated using the ΔΔCt method for the formulation (Api-Cy-13), pectin-coated, apigenin–cyclodextrin complex encapsulated nanoliposomes, in contrast with free apigenin and apigenin encapsulated plain nanoliposomes. Also, a standard drug and a non-treated sample were considered as reference samples. The above experiment was conducted using HCT116 cell lines, pretreated with a 20 ng/mL concentration of verapamil to compare the MDR1 gene expression level both in the presence and absence of verapamil, as a potent Pgp inhibitor [41].

#### 4.1.9. In Vivo Studies

##### Intestinal Pharmacokinetics

Apigenin concentrations in rat plasma were quantified using a validated HPLC method [13]. Briefly, 100 µL of plasma was mixed with 300 µL of acetonitrile containing apigenin, obtained from Sigma Pvt Ltd., Bangalore, India, with (100 µg/mL) as the internal standard for protein precipitation. Samples were vortexed, centrifuged (6000 rpm, 8 min), and then the clear supernatant was evaporated under nitrogen. The residue was reconstituted with 100 µL of methanol:water:acetic acid (68:30.4:1.6, *v*/*v*/*v*) as the mobile phase.

Analysis was carried out on a Shimadzu LC-20AD HPLC system using an Inertsil C18 column (150 × 4.6 mm, 5 µm) at a flow rate of 1 mL/min and detection wavelength of 420 nm. The calibration curve was linear (100–1000 ng/mL; R^2^ = 0.999).

Male Wistar rats (200–250 g) were used with ethical clearance (IAEC No. NCOPS/IAEC/02/2025-26; CPCSEA Reg. No. 2322/PO/Re/S/2024/CPCSEA). The animals were maintained under standard conditions (20 ± 2 °C; 50–60% RH) and fasted for 12 h before dosing. The rats were divided into three groups (n = 4) and orally administered 100 mg/kg of: (i) free apigenin, (ii) plain apigenin nanoliposomes, or (iii) (Api-Cy-13) pectin-coated apigenin–cyclodextrin nanoliposomes.

Blood samples (~250 µL) were collected at 0–48 h via the retro-orbital plexus, centrifuged (5000 rpm, 10 min), and the plasma stored at −20 °C. Pharmacokinetic parameters (Cmax, Tmax, AUC_0_–last, AUC_0_–∞, T½, MRT, AUMC, clearance) were estimated using non-compartmental analysis (Phoenix WinNonlin v6.3, Pharsight, Sunnyvale, CA, USA).

##### Safety Studies

At the end of the pharmacokinetic study, animals were sacrificed, and vital organs, including the heart, lungs, kidneys, and intestine, were excised for histopathological examination. The tissues were carefully trimmed, rinsed with saline, and fixed in 10% neutral buffered formalin (10:1 fixative-to-tissue ratio) for at least 24–48 h. Samples were processed through graded alcohols, embedded in paraffin, sectioned at 4–5 µm, and stained with hematoxylin and eosin (H&E) [42].

Microscopic examination was performed by a qualified pathologist blinded to treatment groups, and observations were recorded using standard diagnostic terminology and semi-quantitative grading according to OECD Test Guideline 407. The histological assessment focused on detecting any cellular or structural abnormalities in the heart, lungs, kidneys, and intestine to evaluate the safety and tissue compatibility of the (Api-Cy-13) formulation.

### 4.2. Statistical Analysis

All treatments were performed in triplicate. Data were expressed as mean ± SD and analyzed statistically using one-way ANOVA, with *p* < 0.05 considered significant.

## Figures and Tables

**Table 1:** Levels followed for strategic design in formulation optimization.

Variable (Independent)	Level Followed		
	(−1)	0	(+1)
β-Cyclodextrin (mg)	40	60	80
Phospholipid (mg)	60	70	80
Methylated Pectin (%)	0.5	0	1

**Table 2:** Formulations using central composite design approach.

	Factor 1	Factor 2	Factor 3
Run	A: β-Cyclodextrin (mg)	B: Phospholipid (mg)	C: Methylated Pectin (%)
1	60	60	0.5
2	60	80	0.5
3	60	70	0.5
4	40	80	1
5	40	60	1
6	80	60	0
7	40	70	0.5
8	60	70	1
9	80	80	1
10	80	60	1
11	40	60	0
12	80	80	0

**Table 3:** Factors vs. experimental responses for parameters of F1-F17, using CCD.

		Factor 1	Factor 2	Factor 3	Response 1	Response 2	Response 3	Response 4	Response 5	Response 6
Std	Run	A: β-Cyclodextrin	B: Phospholipid	C: Methylated Pectin	Drug Loading	Particle Size	Zeta Potential	In Vitro Drug Release in Gastric Simulated pH (at 4 h)	In Vitro Drug Release in Intestine Simulated pH in Absence of Pectinase (Between 4 and 8 h)	In Vitro Drug Release in Intestine Simulated pH in Presence of Pectinase
		mg	Mg	%	%	nm	mV	%	%	%
11	1	60	60	0.5	84	319	−6.11	24	54	72
12	2	60	80	0.5	85	353	−8.5	28	46	74
17	3	60	70	0.5	86	315	−7.11	25	47	72
7	4	40	80	1	82	632	−26	13	49	62
5	5	40	60	1	79	611	−32	11	54	65
2	6	80	60	0	91	155	0.2	45	44	41
9	7	40	70	0.5	82	320	−8.4	19	62	74
14	8	60	70	1	88	697	−24.1	10	51	61
8	9	80	80	1	93	721	−22	12	58	70
6	10	80	60	1	90	770	−19	11	57	68
1	11	40	60	0	81	151	0.12	44	40	43
4	12	80	80	0	92	211	0.3	49	40	41
10	13	80	70	0.5	95	313	−9.5	22	65	75
15	14	60	70	0.5	87	320	−6.9	22	54	74
16	15	60	70	0.5	83	321	−7.23	21	52	73
3	16	40	80	0	82	244	−0.012	61	31	34
13	17	60	70	0	84	158	0.231	51	37	39

**Table 4:** (**a**). ANOVA for quadratic model for particle size zeta potential, and drug loading. (**b**). ANOVA for quadratic model in vitro drug release in gastric simulated pH (at 4 h), in vitro drug release in intestine simulated pH in absence of pectinase (between 4 and 8 h), and in vitro drug release in intestine simulated pH in presence of pectinase.

(a)																		
Factors	Drug Loading (Y1)						Particle Size (Y2)						Zeta Potential (Y3)					
	Sum of Squares	df	Mean Square	F-Value	*p*-Value		Sum of Squares	df	Mean Square	F-Value	*p*-Value		Sum of Squares	df	Mean Square	**F-Value**	***p*** **-Value**	
**Model**	11,925.26	9	1325.03	6.66	0.0103	significant	7.051 × 10^5^	9	78,345.51	216.24	<0.0001	significant	1696.84	9	188.54	47.73	<0.0001	significant
A-β-Cyclodextrin	4724.54	1	4724.54	23.74	0.0018		4494.40	1	4494.40	12.40	0.0097		26.54	1	26.54	6.72	0.0358	
B-Phospholipid	2351.93	1	2351.93	11.82	0.0109		2402.50	1	2402.50	6.63	0.0367		0.0334	1	0.0334	0.0085	0.9293	
C-Methylated Pectin	0.4000	1	0.4000	0.0020	0.9655		6.310 × 10^5^	1	6.3 × 10^5^	1741.62	<0.0001		1536.09	1	1536.09	388.91	<0.0001	
AB	3295.10	1	3295.10	16.55	0.0048		1431.13	1	1431.13	3.95	0.0872		9.61	1	9.61	2.43	0.1628	
AC	0.5000	1	0.5000	0.0025	0.9614		9591.13	1	9591.13	26.47	0.0013		34.48	1	34.48	8.73	0.0213	
BC	2.00	1	2.00	0.0100	0.9230		3916.13	1	3916.13	10.81	0.0133		1.15	1	1.15	0.2909	0.6063	
A^2^	85.79	1	85.79	0.4310	0.5325		53.07	1	53.07	0.1465	0.7133		3.78	1	3.78	0.9566	0.3606	
B^2^	249.94	1	249.94	1.26	0.2994		606.80	1	606.80	1.67	0.2367		0.5607	1	0.5607	0.1419	0.7175	
C^2^	178.34	1	178.34	0.8960	0.3754		30,416.81	1	30,416.81	83.95	<0.0001		46.63	1	46.63	11.81	0.0109	
**(b)**																		
**Factors**	**In Vitro Drug Release in gastric simulated pH (at 4 h) (Y4)**						**In Vitro Drug Release in intestine simulated pH in absence of pectinase (between 4 and 8 h) (Y5)**						**In Vitro Drug Release in intestine simulated pH in presence of pectinase (Y6)**					
	**Sum of Squares**	**df**	**Mean Square**	**F-value**	***p*-value**		**Sum of Squares**	**df**	**Mean Square**	**F-value**	***p*-value**		**Sum of Squares**	**df**	**Mean Square**	**F-value**	***p*-value**	
**Model**	4163.68	9	462.63	69.57	<0.0001	significant	1258.41	9	139.82	15.34	0.0008	significant	3548.38	9	394.26	112.37	<0.0001	significant
A-β-Cyclodextrin	8.10	1	8.10	1.22	0.3062		78.40	1	78.40	8.60	0.0220		28.90	1	28.90	8.24	0.0240	
B-Phospholipid	78.40	1	78.40	11.79	0.0109		62.50	1	62.50	6.85	0.0345		6.40	1	6.40	1.82	0.2189	
C-Methylated Pectin	3724.90	1	3724.90	560.13	<0.0001		592.90	1	592.90	65.03	<0.0001		1638.40	1	1638.40	466.96	<0.0001	
AB	24.50	1	24.50	3.68	0.0964		15.13	1	15.13	1.66	0.2387		24.50	1	24.50	6.98	0.0333	
AC	12.50	1	12.50	1.88	0.2127		0.1250	1	0.1250	0.0137	0.9101		4.50	1	4.50	1.28	0.2947	
BC	40.50	1	40.50	6.09	0.0430		10.13	1	10.13	1.11	0.3270		8.00	1	8.00	2.28	0.1748	
A^2^	17.12	1	17.12	2.58	0.1526		217.02	1	217.02	23.80	0.0018		11.72	1	11.72	3.34	0.1103	
B^2^	23.66	1	23.66	3.56	0.1012		54.25	1	54.25	5.95	0.0448		0.9375	1	0.9375	0.2672	0.6211	
C^2^	149.58	1	149.58	22.49	0.0021		295.39	1	295.39	32.40	0.0007		1345.35	1	1345.35	383.44	<0.0001	

**Table 5:** In vivo pharmacokinetics parameters.

Pharmacokinetics Parameter	Free Apigenin	Plain Apigenin Nanoliposomes	(Api-Cy-13) Apigenin-Cyclodextrin Complex Encapsulated Nano Liposomes with Pectin Coating
**Cmax (ng/mL)**	8.7 ± 0.54	24.2 ± 0.53	33.2 ± 0.65
**Tmax (h)**	3.0 ± 0.4	4.0 ± 0.5	6.0 ± 0.25
**AUC 0-last (ng·h/mL)**	30.3 ± 2.7	120.1 ± 7.9	199.85 ± 6.90
**T1/2 (h)**	0.86 ± 0.08	1.50 ± 0.15	1.94 ± 0.07
**MRT (h)**	3.22 ± 0.25	4.27 ± 0.30	5.56 ± 0.29
**AUMC (ng·h^2^/mL)**	97.5 ± 8.4	512.3 ± 47.9	1111.1 ± 44.0
**AUC 0-∞ (ng·h/mL)**	31.05 ± 2.6	124.85 ± 9.0	225.91 ± 13.4
**Clearance (L/h/kg) (dose = 1)**	0.032 ± 0.003	0.008 ± 0.001	0.0044 ± 0.0005

## Data Availability

The original contributions presented in this study are included in the article/Supplementary Material. Further inquiries can be directed to the corresponding author.

## Supplementary Materials

The following supporting information can be downloaded at: https://www.mdpi.com/article/10.3390/gels11110873/s1, Figure S1: Swelling index for the formulation (Api-Cy-13) at different experimental conditions.; Table S1: Regression analysis of formulation characterization parameters in gastric simulated pH at 4 h, in vitro drug release in intestine stimulated pH in absence of pectinase between 4–8 h and in vitro drug release in intestine stimulated pH in presence of pectinase.
