# Supplementary material for: Oral Delivery of a GI-Stable Apigenin–Cyclodextrin Complex via Pectin-Coated Nanoliposomes In Situ Gel: A DoE-Optimized Targeted Colon Cancer Therapy by Modulating Gut Drug Sensitivity
Source: Gels. 2025 Oct 31;11(11):873. doi: 10.3390/gels11110873 (PMC12652451; doi:10.3390/gels11110873)
Supplement: Supplementary file 1 [file gels-11-00873-s001.zip › gels-3931701-supplementary.pdf]

gels

MDPI

Oral Delivery of a GI-Stable Apigenin–Cyclodextrin Complex via Pectin-Coated Nanoliposomes *In Situ* Gel: A DoE Optimized Targeted Colon Cancer Therapy by Modulating Gut Drug Sensitivity

Moumita Dhara [1,*], Kusum Devi Vemula [2], Ziaul Karim [2] and Anoop Narayan V [3], Tanvi Shetty [1] and Anushree Prakash M [1].

Table S1: Regression analysis quantified the contributions of formulation factors to release behavior with predicting p-value and R statistics. The predictive equations revealed strong correlation coefficients, validating the robustness of the CCD approach. The release pattern was characterized by minimal drug liberation in gastric pH, sustained release under intestinal pH without enzymatic activity, and accelerated release upon exposure to pectinase, demonstrating the enzyme-triggered responsiveness of the system.

Table S1. Regression analysis of formulation characterization parameters in gastric simulated pH at 4hr, *in vitro* drug release in intestine stimulated pH in absence of pectinase between 4-8Hr and *in vitro* drug release in intestine stimulated pH in presence of pectinase.

Source	Sequential p-value	Lack of Fit p-value	Adjusted R^2	Predicted R^2	Remarks
Model Fit Summary: Drug Loading (Y1)					
Linear	0.0170	0.0076	0.4232	0.0029	
2FI	0.0492	0.0117	0.6463	-0.3983	Suggested
Quadratic	0.1347	0.0155	0.7609	0.2062	Suggested
Model Fit Summary: Particle Size (Y2)					
Linear	< 0.0001	0.0016	0.8787	0.8178	
2FI	0.4709	0.0015	0.8761	0.5548	
Quadratic	< 0.0001	0.0202	0.9918	0.9680	Suggested
Model Fit Summary: Zeta Potential (Y3)					
Linear	< 0.0001	0.0019	0.8845	0.8199	
2FI	0.3297	0.0019	0.8918	0.5848	
Quadratic	0.0137	0.0050	0.9634	0.7758	Suggested
Model Fit Summary: In Vitro Drug Release in gastric simulated pH (at 4hr) (Y4)					
Linear	< 0.0001	0.1138	0.8834	0.8273	
2FI	0.5198	0.1036	0.8779	0.5811	
Quadratic	0.0025	0.4025	0.9747	0.8572	Suggested
Model Fit Summary: In Vitro Drug Release in intestine stimulated pH in absence of pectinase (between 4-8Hr) (Y5)					
Linear	0.0123	0.2201	0.4523	0.3069	
2FI	0.9272	0.1723	0.3187	-0.2395	
Quadratic	0.0011	0.7296	0.8897	0.8227	Suggested
Model Fit Summary: In Vitro Drug Release in intestine stimulated pH in presence of pectinase (Y6)					
Linear	0.0367	0.0058	0.3458	0.1225	
2FI	0.9766	0.0043	0.1661	-1.5705	
Quadratic	< 0.0001	0.1913	0.9843	0.9399	Suggested

 https://doi.org/10.3390/xxxxx

Figure S1. graphically represents the pH-dependent swelling behavior of the lyophilized pectin-coated nanoliposome formulation (Api-Cy-13). The plot illustrates a distinct difference in the swelling index (SI) under simulated gastrointestinal conditions. At acidic pH (SGF, pH 1.2), the formulation shows a pronounced swelling of approximately 90%, signifying robust gel formation due to protonation of carboxyl groups in pectin. Conversely, at near-neutral pH (SIF, pH 6.8), the SI markedly decreases to below 20%, indicating matrix relaxation and partial disintegration of the gel network. This graphical trend confirms the pH-responsive nature of the pectin coating for protecting the encapsulated complex in the gastric environment while promoting drug release in the intestinal region. The clear visual distinction in swelling behavior across pH conditions reinforces the formulation's potential for controlled, site-specific drug delivery.

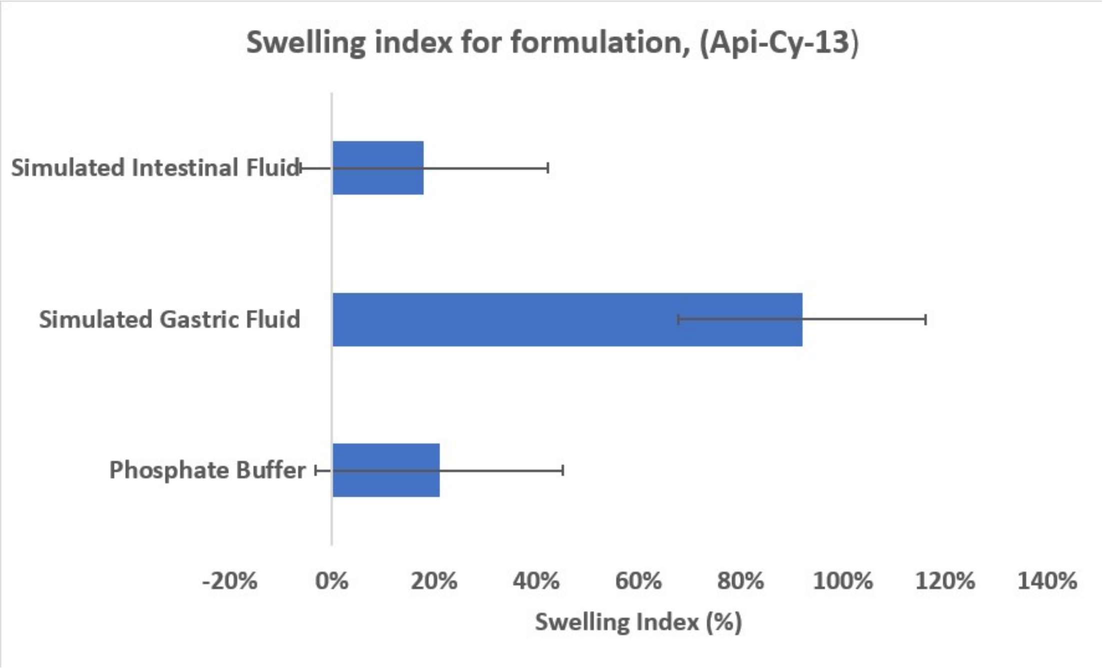

Figure S1. Swelling index for the formulation (Api-Cy-13) at different experimental conditions.
